# DIABETES OCCURRENCE, RISK FACTORS, AND LIFESTYLE PATTERNS IN OLDER ADULTS ATTENDING SENIOR CENTERS

**DOI:** 10.1093/geroni/igad104.2580

**Published:** 2023-12-21

**Authors:** Laurie Ruggiero, Elizabeth Orsega-Smith, Sophia Kayatta

**Affiliations:** University of Delaware, Newark, Delaware, United States; University of Delaware, Newark, Delaware, United States; University of Delaware, Newark, Delaware, United States

## Abstract

Diabetes risk and rates increase across age with prevalence rates of diagnosed diabetes of 24% and estimated pre-diabetes rates of 49% in adults 65 or older (2017-2020; CDC, 2020) underscoring the importance of understanding diabetes risk factors and lifestyle behaviors in older adults. Senior centers offer a unique opportunity to reach older adults to deliver health promotion interventions. The study purpose was to examine diabetes/pre-diabetes occurrence, risk factors, lifestyle behaviors, and preferences for promoting healthy lifestyles to reduce diabetes risk in older adults (>60). An anonymous cross-sectional self-report survey was administered in senior centers. It included questions regarding occurrence of diabetes, diabetes risk factors, and hypertension; self-reported health status; lifestyle behavior patterns; cognitive function; and social engagement. Open-ended questions obtained information on diabetes risk awareness and preferences for healthy lifestyle interventions delivered in senior centers. The sample included 159 adults: average age of 76 years (range: 60-96), 77% female, 83% White, and 14% African American. Reported health related patterns include: 89% rated health as good-excellent; 74% were at high risk for pre-diabetes; 64% had hypertension; and 20% were told by provider of diabetes and 32% pre-diabetes. Reported lifestyle patterns included: 73% get regular physical activity, 53% eat 5 daily fruits/vegetables, 7% smoke, and average 7 hours of sleep/night. The large proportion at high risk for pre-diabetes underscores the importance of focusing on diabetes prevention in older adults and potential for delivering healthy lifestyle interventions in senior centers. Presentation will describe the quantitative and qualitative results and implications for lifestyle interventions.

